# Co‐occurrence of collagenous gastrointestinal disease in siblings in early childhood: New insight into a rare condition

**DOI:** 10.1002/jpr3.12097

**Published:** 2024-06-14

**Authors:** Taryn L. Luitingh, Jessica Ng, Kathleen H. McGrath

**Affiliations:** ^1^ Department of Neurodevelopment and Disability The Royal Children's Hospital Melbourne Victoria Australia; ^2^ Department of Anatomical Pathology The Royal Children's Hospital Melbourne Victoria Australia; ^3^ Department of Gastroenterology and Clinical Nutrition The Royal Children's Hospital Melbourne Victoria Australia; ^4^ Department of Paediatrics The University of Melbourne Melbourne Victoria Australia

**Keywords:** gastroenterology, pathology, pediatrics

## Abstract

Collagenous gastrointestinal (GI) disease is a rare inflammatory condition characterized by subepithelial collagen deposition and inflammatory cell infiltrates of the GI mucosa, which typically occurs in the stomach in children. There are few published reports of more extensive involvement in children, and descriptions of familial involvement are rare, with no previous reported cases of affected siblings in early childhood. We describe two siblings with contrasting medical backgrounds, who were simultaneously diagnosed with collagenous GI disease in early childhood. Both children demonstrated gastric and colonic involvement on serial endoscopies, however, had distinct patterns of clinical presentation, disease course, and histological progression, providing new insights into the pediatric phenotype of collagenous GI disease and further, its relationship to microscopic colitis. Although rare, this condition should be considered as a differential in children presenting with severe or refractory iron deficiency anemia, chronic non‐bloody watery diarrhea, or unexplained nonspecific chronic abdominal pain.

## 1 INTRODUCTION

Collagenous gastrointestinal (GI) disease is a rare inflammatory condition characterized by subepithelial collagen deposition and thickening of the collagen band to greater than 10 µm, with associated inflammatory cell infiltrates of the GI mucosa.^1^, ^2^ Childhood disease is commonly associated with collagen deposition in the stomach and severe iron deficiency anemia, with or without abdominal pain or vomiting^3^, ^4^, ^5^; compared to adult involvement with more extensive GI collagen deposition and nonspecific chronic diarrhea which is usually non‐bloody.^1^ Pathophysiology is poorly understood. Most published cases are in adults, with female predominance^1^, ^6^ and observations of familial involvement involving children are rare.^7^ We report the co‐occurrence of collagenous GI disease in siblings in early childhood, bringing new insight into the clinical features and disease course of this condition.

## 2 CASE REPORT (PATIENT 1)

**Figure 1:**
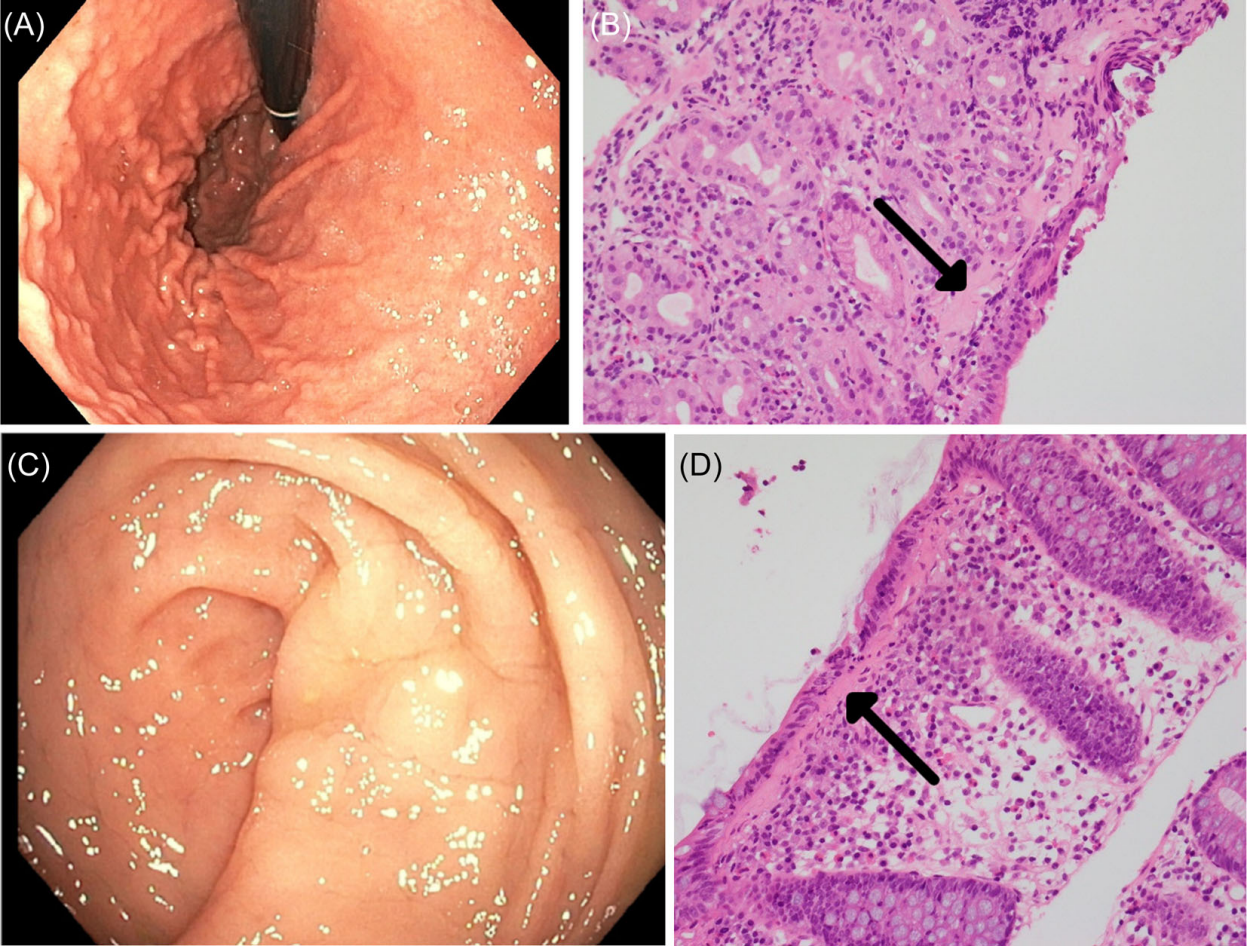
Initial endoscopic and histopathological findings in patient 1 (May 2022). Clockwise rotation from top left: (A) erythema and nodularity with hypertrophic folds in gastric body; (B) subepithelial collagen thickening and clusters of plasma cells in lamina propria of stomach—H&E stain (×100); (C) mild nodularity in rectum; (D) global thickening of the subepithelial collagen plate and mild to moderate plasma cell infiltration in lamina propria of colon—H&E stain (×100). H&E, hematoxylin and eosin.

Patient 1 presented at 8 years of age (May 2021) with pallor and lethargy in the setting of severe iron deficiency anemia [hemoglobin (Hb) 60 g/L (reference range: 115–155 g/L), ferritin <1 µg/L (reference range: 11–109 µg/L)]. She had an unremarkable past medical history and no regular medications. An intravenous iron infusion was given, however, 6 months later, she had ongoing anemia with new symptoms of abdominal pain, nausea, and non‐bloody diarrhea and was referred to gastroenterology. Further investigations at this time showed elevated erythrocyte sedimentation rate (ESR) 39 mm/h (reference range: 2–10 mm/h), normal C‐reactive protein (CRP), normal celiac serology (on adequate dietary gluten intake), and normal stool microscopy, culture, and calprotectin [12 µg/g (normal value < 50 µg/g)]. Upper GI endoscopy and colonoscopy were performed in May 2022 and revealed collagenous gastritis and colitis (Table 1; Figure 1). Following endoscopy, a weaning course of oral prednisolone was prescribed (starting dose 1 mg/kg/day). Six‐weeks later at the time of completion of the course, she reported improved symptoms with normalization of serum inflammatory markers.

**Table 1:** Summary of serial endoscopic findings.

	May 2022	February 2023	April 2023	September 2023
*Patient 1*				
Endoscopic appearance				
Stomach	Marked erythema and nodularity in gastric body and mild involvement of antrum.		Marked nodularity in gastric body with erythema in antrum.	
Small intestine	Normal duodenum and ileum.		Mild nodularity in duodenum.	
Colon	Mild nodularity in rectum, rest normal.		Normal	
Histology (no. of biopsies)				
Stomach	Prominent thickening of subepithelial collagen plate^a^ in gastric body (3) and antrum (2); clusters of plasma cells in lamina propria and eosinophils.		Gastric body (3): prominent subepithelial collagen thickening,^a^ patchy lamina propria lymphocytes/eosinophils. Antrum (2): patchy mild thickening of subepithelial collagen [trichrome stain], lamina propria plasma cells.	
Small intestine	Normal duodenum (4) and ileum (2).		Normal duodenum (2) and ileum (3).	
Colon	Thickening of subepithelial collagen plate^a^ in all biopsies (11)^b^; mild to moderate increase in plasma cells in lamina propria; mild mucosal irregularity.		Thickening of subepithelial collagen plate^a^ in all biopsies (10) (seen on trichrome stain only in ascending colon and rectum) with patchy irregularities to crypt spacing. Increased plasma cells in lamina propria in sigmoid colon (2).	
*Patient 2*				
Endoscopic appearance				
Stomach	Normal	Patchy erythema in antrum and gastric body, prominent gastric folds		Marked erythema and nodularity in gastric body; mild involvement of antrum.
Small intestine	Normal	Normal duodenum		Normal duodenum
Colon	Normal	Normal^c^		Loss of vascular pattern, mild nodularity and edematous mucosa.^c^
Histology (no. of biopsies)				
Stomach	Normal gastric body (2) and antrum (2)	Marked thickening in subepithelial collagen plate^a^ in 2 of 3 gastric body biopsies, with patchy mild increase in lamina propria lymphocytes		Gastric body (2): small number eosinophils and neutrophils focally associated with acute pititis and pit abscesses in lamina propria. No subepithelial collagen plate thickening.^a^ Antrum (1): normal.
Small intestine	Normal duodenum (2) and ileum (2)			
Colon	Thickening of subepithelial collagen plate^a^ in all colonic biopsies (10)^b^; mild epithelial lymphocytic infiltrate, mild to moderate increase in plasma cells in lamina propria; mild mucosal irregularity.	Mild increase in lymphocytes in epithelium and lamina propria of rectum (3) with associated focal degeneration of surface epithelium. No obvious thickening of subepithelial collagen plate.^a^, ^c^		Patchy minor crypt shortening in rectum (2), mild increase in eosinophils and scattered neutrophils in lamina propria. No thickening of subepithelial collagen plate.^a^, ^c^

a Hematoxylin and eosin and trichrome stains performed.

^b^ Colonic biopsies (×1–3 taken) from all levels: cecum, ascending colon, transverse colon, descending colon, sigmoid colon, and rectum.

^c^ Flexible sigmoidoscopy only.

Endoscopic reassessment was planned but delayed due to the coronavirus disease (COVID)‐19 pandemic. In December 2022, she had a recurrence of abdominal pain and mild increase in stool frequency, with a rise in her inflammatory markers [CRP 18 mg/L (normal < 5 mg/L) and ESR 19 mm/h]. She commenced a proton pump inhibitor with symptomatic benefit. She was referred to Allergy and Immunology outpatient clinic for assessment and mild immunoglobulin A (IgA) deficiency 0.26 g/L (reference range: 0.51–2.97 g/L) was identified, with otherwise normal immune function tests (full blood count, lymphocyte subsets, naïve T cells, immunoglobulins, regulatory T cells, tetanus antibodies, interferon antibody testing) and normal serum albumin.

Endoscopic reassessment (April 2023) showed persistent collagenous GI disease (Table 1) despite normal serum inflammatory markers (CRP, ESR, platelet count), and ferritin. She reported intermittent abdominal pain and recent increased frequency of stooling at this time. Oral budesonide delayed‐release capsules were commenced (off‐label use; starting dose 6 mg daily), and proton pump inhibitor continued. Seven‐weeks later, her diarrhea resolved, and abdominal pain improved. Budesonide was weaned over subsequent weeks in a stepwise approach at which point she started to complain of abdominal pain and nausea after eating and was recommenced on 3 mg daily budesonide, this time administered as open‐capsule contents in apple sauce. Following this, GI symptoms resolved, and she remained asymptomatic with further weaning and cessation of budesonide.

## 3 CASE REPORT (PATIENT 2)

Patient 2 is the younger brother of our first case and was initially seen by gastroenterology at 3 years‐of‐age (March 2022) for review of chronic watery diarrhea and weight loss over 12 months but worsened since COVID‐19 infection in October 2021. In contrast to his sister, he had a complex medical history: prematurity (35 weeks gestation), dichorionic diamniotic twins [twin deceased], de novo Q motif and Sec7 domain 2 (IQSEC2)‐related disorder, epilepsy, glucose‐6‐phosphate dehydrogenase deficiency, gastro‐esophageal reflux disease, and long‐term enteral feeding. IQSEC2‐related disorder is a rare genetic progressive syndrome with characteristic features including severe intellectual disability, hypotonia, moderate to severe delayed psychomotor development, and refractory epilepsy.^8^ Medications included omeprazole, topiramate, sodium valproate, clobazam, domperidone, vitamin C/D/zinc supplements. Patient 1 and 2 live with their parents, both of whom are well and nonconsanguineous, with a paternal history of smoking. There is a history of Crohn's disease in paternal grandmother but no other autoimmune conditions. Patient 2 had a twin sister who died at 2 years of age from a rare autosomal recessive leukodystrophy.

Inflammatory markers (CRP, ESR, and platelet count), Hb, ferritin, celiac serology (on small amounts of daily gluten intake via pureed solid food), fecal microscopy, culture, elastase, and *Clostridium difficile* were all normal at the time of initial review (March 2022), however, he had elevated serum inflammatory markers in November 2021 [CRP 63 mg/L (normal < 5 mg/L), ESR 19 mm/h (reference range: 2–10 mm/h)] following COVID‐19 infection. Stool calprotectin was elevated on three occasions before initial review: December 2021 (951 μg/g), January 2022 (594 μg/g), and February 2022 (1195 μg/g) [normal value < 50 μg/g]. Notably, his sister had not been diagnosed with collagenous GI disease at this point. He was placed on the waiting list for endoscopy however given the onset of his symptoms following recurrent respiratory tract infections and associated antibiotic courses, and in the absence of a clear infective cause, a decision was made to treat for presumed small intestinal bacterial overgrowth (SIBO). This was a clinical diagnosis given the challenges and associated limitations of diagnostic testing with breath tests in this age group and before opportunity to obtain a small intestinal aspirate at the time of endoscopy. He responded well to a 1‐week course of amoxicillin, metronidazole, and nystatin, with short‐term clinical improvement.

**Figure 2:**
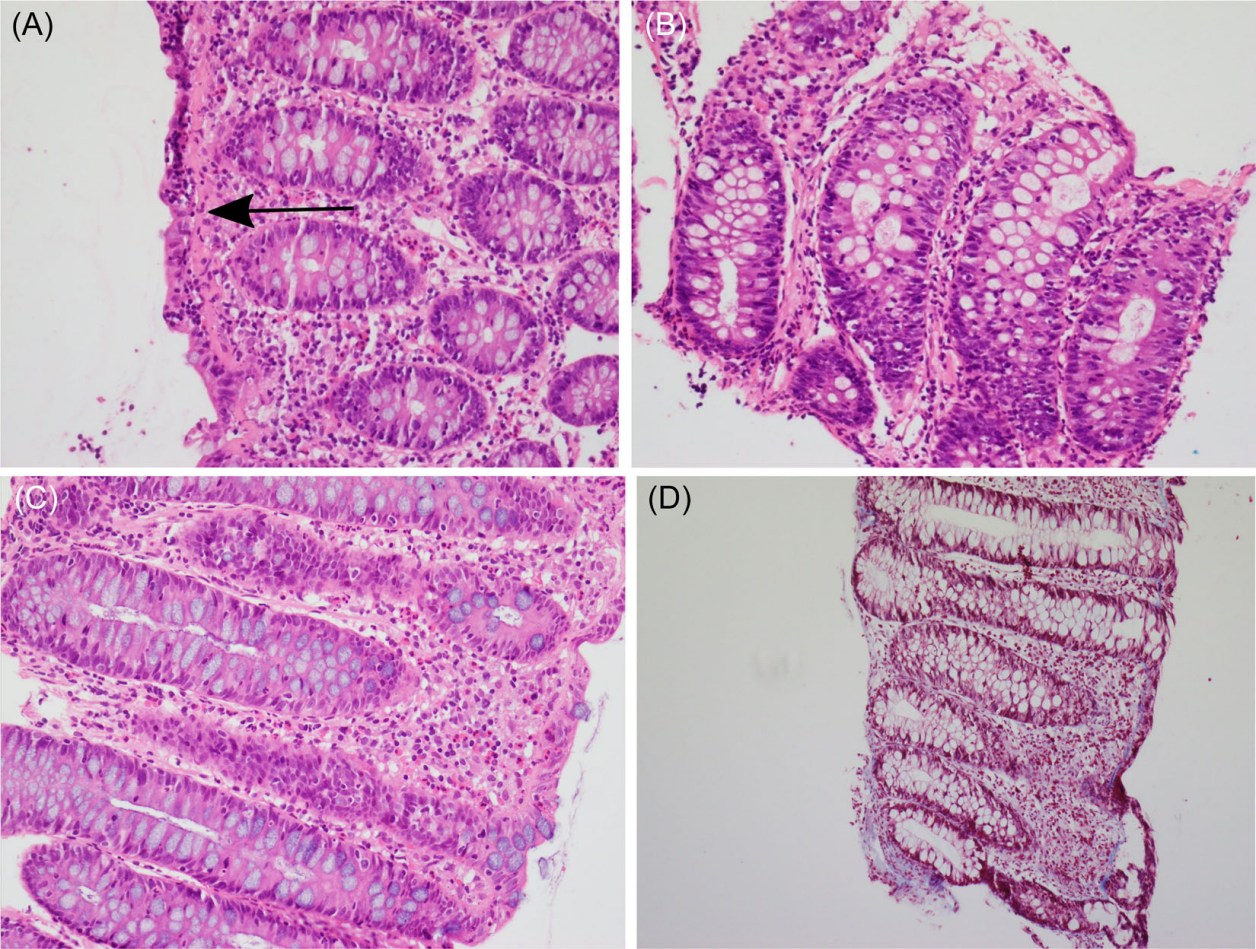
Serial histopathological findings in rectum in patient 2. Clockwise rotation from top left: (A) (May 2022) collagenous colitis with thickening of the subepithelial collagen plate and mild to moderate plasma cell infiltration in lamina propria—H&E stain (×100); (B) (February 2023) lymphocytic colitis with mild increase in lymphocytes in the epithelium and lamina—H&E stain (×100); (C) (September 2023) mild colitis with focal neutrophils, mild increase in lamina propria eosinophils and no subepithelial collagen plate thickening—H&E stain (×100), confirmed on trichrome stain (×100) (D). H&E, hematoxylin and eosin.

Upper GI endoscopy and colonoscopy were performed on the same day as his sister (May 2022) and showed histological findings of collagenous colitis (Table 1; Figure 2). Following endoscopy, a 6‐week weaning course of oral prednisolone was prescribed (starting dose 1 mg/kg/day). At the time of completion, his stool frequency had improved, but not consistency and further SIBO treatment was prescribed with a 2‐month course of a cycling antibiotic regimen (amoxicillin and metronidazole) with probiotic. Symptoms relapsed following COVID‐19 infection (September 2022) with increased watery diarrhea and soluble fiber was added to his formula and loperamide commenced. Around this time, he was reviewed in the Allergy and Immunology clinic at the same time as his sister and also had mild IgA deficiency of 0.18 g/L (reference range: 0.22–2.20 g/L) identified in the context of a borderline low IgM level 0.37 g/L (reference range: 0.40–1.40 g/L) but normal IgG, serum albumin and otherwise normal immune function testing (full blood count, lymphocyte subsets, naïve T cells, immunoglobulins, regulatory T cells, tetanus antibodies, interferon antibody testing).

Endoscopic reassessment at the time of elective percutaneous endoscopic gastrostomy (PEG) insertion (February 2023) showed new onset collagenous gastritis and evolution to lymphocytic colitis in rectal biopsies (Table 1; Figure 2). He was commenced on budesonide delayed‐release capsules (open capsule contents in apple sauce; starting dose 3 mg daily) with subsequent significant improvement in his watery diarrhea and excellent growth over 8 weeks. Budesonide was weaned over 4 weeks, then ceased. Subsequent biopsies at time of elective change of initial PEG to balloon gastrostomy device all showed resolution of collagen plate thickening (Table 1).

## 4 DISCUSSION

Collagenous GI disease is considered rare, particularly in children. Familial links have been observed in adult populations across siblings^9^, ^10^ and parent–child,^7^, ^10^ however, to our knowledge, affected siblings in childhood have not been reported. This report provides some new insights into collagenous GI disease in children. First, our patients had diverse medical histories, with no clear common precipitant identified, prompting the need for further consideration of genetic risk factors in the pathogenesis of this condition as well as possible household environmental influences through larger population cohort studies.

Second, we observed disease evolution on serial endoscopies, providing further insight into disease course in children and adding to the limited existing literature base. A Swedish cohort study of pediatric collagenous gastritis (*n* = 15) observed persistent collagenous gastritis in all patients with follow‐up endoscopies (11/15), however, no new pathology, and only one patient with concurrent collagenous colitis.^5^ A French national cohort study of pediatric collagenous gastritis (*n* = 12) reported no patients with co‐existing collagenous colitis (nonspecific chronic inflammation present on colonic biopsies in three patients).^3^ Both of our patients had co‐existing collagenous gastritis and colitis which persisted or evolved over time, despite reported clinical improvement with treatment, with collagen deposition up to 50 μm thick in the most affected area. Patient 2 had apparent evolution of histological features of collagenous GI disease with no thickening of the subepithelial collagen plate seen on his second and third colonoscopy biopsies (Table 1), posing question as to whether this reflects natural history of the condition or what extent this was influenced by treatment. However, factors including patchy involvement of inflammatory changes or collagen thickening, as well as sampling bias relating to the number and distribution of biopsies taken may influence findings, thus should be taken into consideration.

Serum inflammatory markers were elevated in both patients at different time points, varying from previous reports of pediatric collagenous gastritis^3^, ^5^; although it is relevant to note that both of our patients had colonic involvement which is unusual in children and the majority of children described in these previous pediatric case series had isolated collagenous gastritis. However, overall, serum inflammatory markers seemed to be unreliable markers of disease activity or treatment response in our patients and further, may have been influenced by recurrent intercurrent infections in Patient 2. Collagenous GI disease is a histological diagnosis. Endoscopic features such as nodularity in the stomach may be difficult to distinguish from other differentials, such as *Helicobacter pylori* gastritis (although nodularity tends to predominantly affect the body of the stomach in collagenous disease compared to antral predominance in *H. pylori* gastritis). The exact role of upper GI endoscopy and/or colonoscopy in evaluating treatment response and monitoring progress requires careful consideration, particularly given the paucity of literature to support standardized therapeutic decision‐making in children, and further research is required. Management tends to be extrapolated from adult studies. Corticosteroids, in particular budesonide, have shown clinical efficacy in collagenous colitis,^2^ which was observed in our patients. Treatment options in collagenous gastritis in children are less clear, with iron supplementation, proton pump inhibitors, and steroids being the most described, as used in our patients, but reported outcomes are variable.^3^, ^4^

Collagenous GI disease should be considered in children with unexplained chronic GI symptoms, particularly severe iron deficiency anemia, which may indicate collagenous gastritis or chronic watery diarrhea in collagenous colitis; however, involvement beyond the stomach is rare in children. This report heralds new insights into this rare condition and dedicated larger cohort follow‐up studies are needed to improve understanding of natural history and patient outcomes.

## AUTHOR CONTRIBUTIONS

Kathleen McGrath and Taryn Luitingh conceptualized and designed the study, collected data, drafted the initial manuscript, and critically reviewed and revised the manuscript. Jessica Ng collected data, contributed to the initial draft, and critically reviewed and revised the manuscript. All authors approved the final manuscript as submitted and agree to be accountable for all aspects of the work.

## CONFLICT OF INTEREST STATEMENT

The authors declare no conflict of interest.

## ETHICS STATEMENT

Written informed consent for this case report and its publication was obtained from the patients' parents and has been archived.
